# Supplementary material for: Primary infection with Zika virus provides one-way heterologous protection against Spondweni virus infection in rhesus macaques
Source: Sci Adv. 2023 Jun 30;9(26):eadg3444. doi: 10.1126/sciadv.adg3444 (PMC10313173; doi:10.1126/sciadv.adg3444)
Supplement: Supplementary file 1 — Figs. S1 to S3 Table S1 [file sciadv.adg3444_sm.pdf]

Science Advances
AAAS

Supplementary Materials for

Primary infection with Zika virus provides one-way heterologous protection against Spondweni virus infection in rhesus macaques

Anna S. Jaeger *et al.*

Corresponding author: Matthew T. Aliota, mtaliota@umn.edu

Sci. Adv. **9**, eadg3444 (2023)
DOI: 10.1126/sciadv.adg3444

This PDF file includes:

Figs. S1 to S3
Table S1

Fig. S1

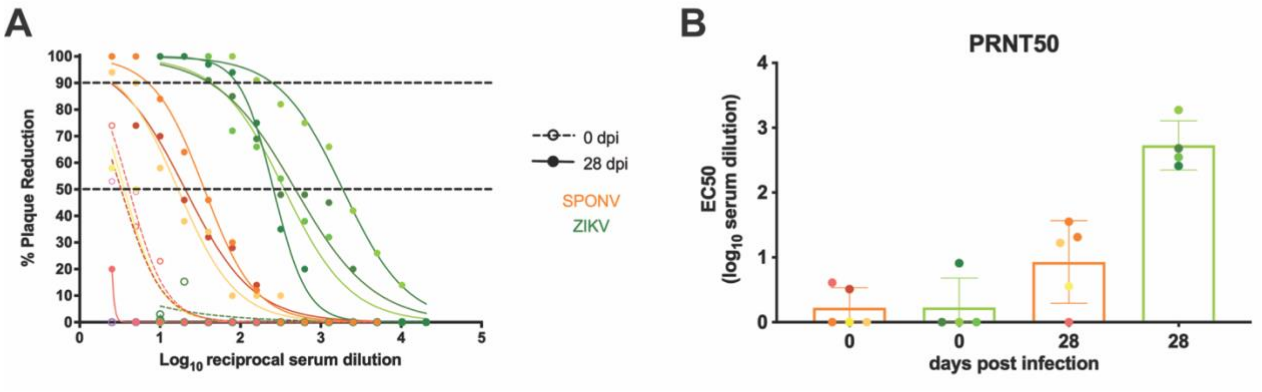

Fig. S1. SPONV and ZIKV neutralization in cynomolgus macaques. A. SPONV (orange) and ZIKV-DAK (green) neutralization curves for cynomolgus macaques 0 (open symbols) and 28 (closed symbols) days post infection. Dotted lines indicate 90% and 50% plaque reduction. **B.** The EC50 neutralization titers for SPONV and ZIKV-DAK.

Fig. S2

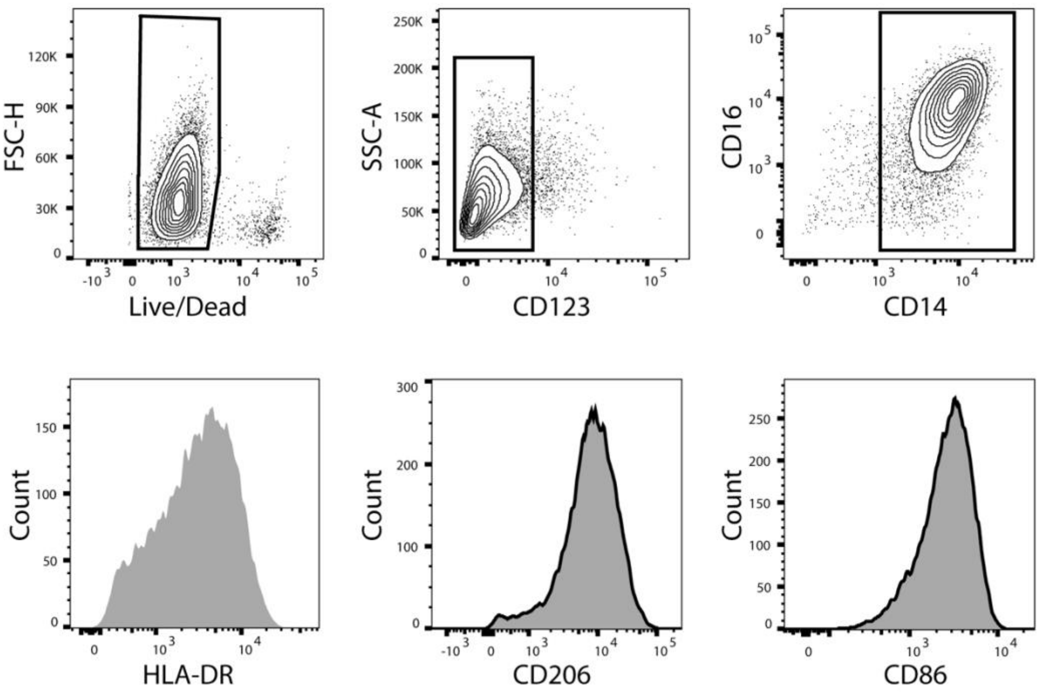

Fig. S2. Flow cytometry analysis of PBMC-derived macrophages. Surface marker expression of PBMC-derived macrophages from cynomolgus macaques were analyzed by flow cytometry to confirm successful differentiation. Cells had high surface expression levels of CD14, CD16, HLA-DR, CD206, and CD86; the dendritic cell marker CD123 was not expressed. Abbreviations: FSC-H (forward scatter-height), SSC-A (side scatter-area).

Fig. S3

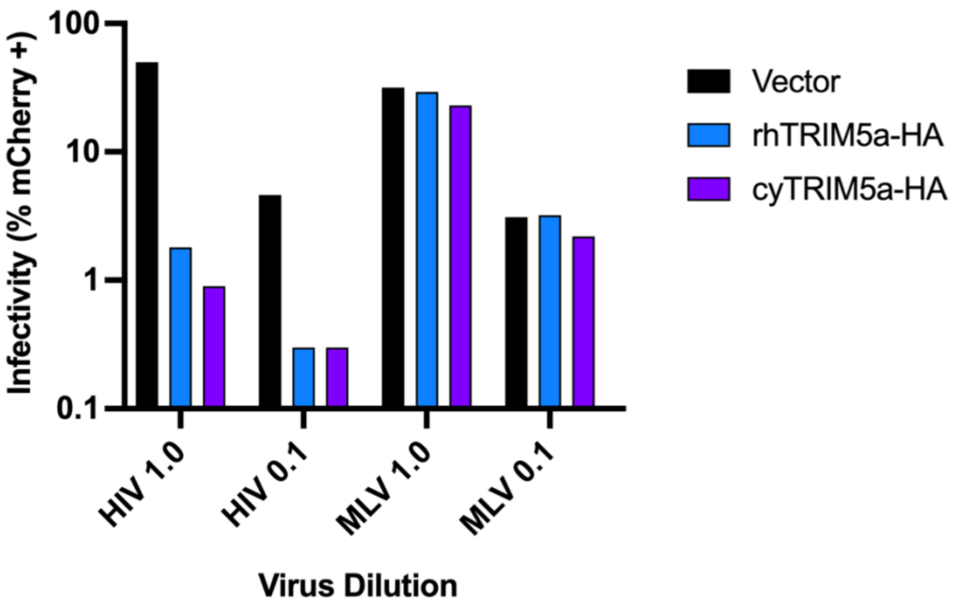

Fig. S3. Macaque TRIM5a inhibits HIV, but not MLV. HEK293 cells transduced as indicated (vector, rhTRIM5a-HA, cyTRIM5a-HA) were infected with single cycle HIV-1 or murine leukemia virus (MLV) mCherry reporter viruses at approximate MOI/cell of 1.0 and 0.1. After 48 hours, % of cells that were mCherry positive was determined by flow cytometry. HIV-1 was restricted by both rhesus and cynomolgus TRIM5-alpha whereas MLV was unaffected.

Table S1: Macaque Demographics

Cohort	Species	Animal ID	Sex	Origin	Age at time of inoculation (yrs)
SPONV/ SPONV	cynomolgus	047-101	M	Chinese	7.0
		047-102	M	Chinese	5.7
		047-103	M	Mauritian	6.8
		047-104	M	Chinese	6.9
		047-105	M	Chinese	5.2
ZIKV-DAK/ SPONV	cynomolgus	047-106	M	Chinese	7.7
		047-107	M	Mauritian	5.8
		047-108	M	Chinese	6.8
		047-109	M	Chinese	6.1
SPONV/ ZIKV-DAK	rhesus	051-101	M	Indian	13.2
		051-102	F	Indian	11.8
		051-103	M	Indian	5.0
		051-104	F	Indian	5.0
ZIKV-DAK/ SPONV	rhesus	051-105	F	Indian	4.8
		051-106	M	Indian	22.2
		051-107	M	Indian	5.2
